# Supplementary material for: High internal phase emulsions gel ink for direct-ink-writing 3D printing of liquid metal
Source: Nat Commun. 2024 Jun 5;15:4806. doi: 10.1038/s41467-024-48906-w (PMC11153652; doi:10.1038/s41467-024-48906-w)
Supplement: Supplementary file 1 — Supplementary Information [file 41467_2024_48906_MOESM1_ESM.pdf]

Supplementary Information

High Internal Phase Emulsions Gel Ink for Direct-Ink-Writing 3D Printing of Liquid Metal

Zewen Lin[1], Xiaowen Qiu[1], Zhouqishuo Cai[1], Jialiang Li[1], Yanan Zhao[1], Xinping Lin[1], Jinmeng Zhang[1], Xiaolan Hu[1*], Hua Bai[1,2*]

1 College of Materials, Xiamen University, Xiamen 361005, PR China

2 Innovation Laboratory for Sciences and Technologies of Energy Materials of Fujian Province (IKKEM), Xiamen, China

** Corresponding author*

E-mail addresses: baihua@xmu.edu.cn, xlhu@xmu.edu.cn

Table of content

Supplementary Figures

Supplementary Fig. 1 | Transmitted microscope images of LM-HIPEG.

Supplementary Fig. 2 | SEM/EDS of LM-HIPEG.

Supplementary Fig. 3 | TEM/EDS of LM-HIPEG.

Supplementary Fig. 4 | XPS spectra of LM-HIPEG.

Supplementary Fig. 5 | State of liquid metal after shearing in water in an air environment.

Supplementary Fig. 6 | The force curves of AFM tip pressing on EGaIn droplets.

Supplementary Fig. 7 | Modulus as a function of shear stress of Carbopol hydrogel.

Supplementary Fig. 8 | The impact of ion concentration on the rheological behavior of Carbopol hydrogels and LM-HIPEG

Supplementary Fig. 9 | Droplet size distribution of LM-HIPEG.

Supplementary Fig. 10 | The relationships between the EGaIn volume fraction and both the energy storage modulus and the yield stress.

Supplementary Fig. 11 | The 3ITT curve of 82.5% LM-HIPEG

Supplementary Fig. 12 | Viscosity as a function of shear rate of Carbopol gel.

Supplementary Fig. 13 | Quantify Carbopol Thickness.

Supplementary Fig. 14 | Properties of the 82.5% LM-HIPEG ink after extrusion

Supplementary Fig. 15 | Images of LM-HIPEG with different volume fraction.

Supplementary Fig. 16 | Compared to common polymer materials.

Supplementary Fig. 17 | The thermogravimetric curve of LM-HIPEG.

Supplementary Fig. 18 | Morphological changes of printed objects over time.

Supplementary Fig. 19 | Images of EGaIn droplets on different polymeric substrates.

Supplementary Fig. 20 | The 2000 cycles of resistance variation in a printed line.

Supplementary Fig. 21 | SEM images of LM-HIPEG after solidification.

Supplementary Fig. 22 | Shapes of printed cube before, during, and after solidification.

Supplementary Fig. 23 | The merging of droplets on printed lines.

Supplementary Fig. 24 | 3D network formed by alternating printing of LM-HIPEG with PDMS/PTFE.

Supplementary Fig. 25 | Top-view of the objects printed with alternating materials.

Supplementary References

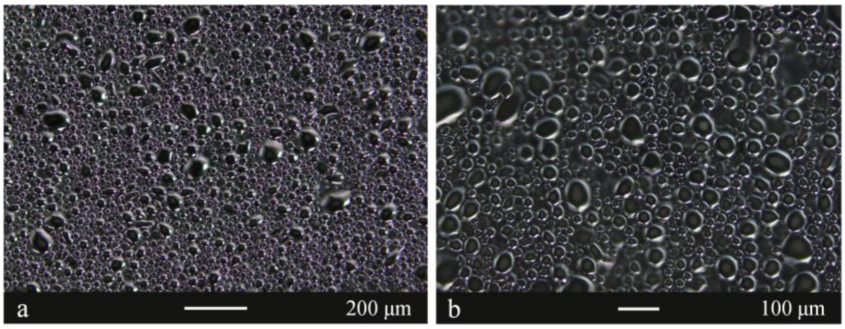

Supplementary Fig. 1 │a-b Transmitted microscope images of LM-HIPEG at different magnification levels.

From Supplementary Fig. 1, it can be observed that LM-HIPEG exhibits a distinct features of high internal phase emulsion, with close contact between liquid metal droplets. The droplets exhibit a non-spherical shape, and the interfaces between the droplets are clearly visible.

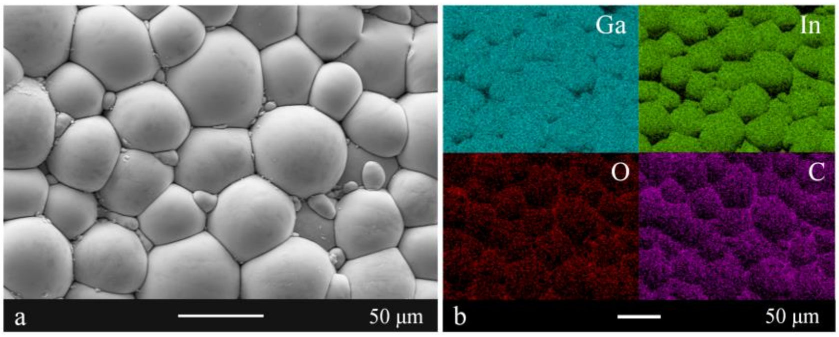

Supplementary Fig. 2 | a SEM image of LM-HIPEG. **b** Energy Dispersive Spectroscopy (EDS) of the selected region in **a**.

From Supplementary Fig. 2a, it can be observed that the liquid metal droplets are uniformly encapsulated by the membrane structure, forming polyhedral shapes and exhibiting the close packing. The droplets are in full contact with each other, separated only by the outer membrane structure. According to Supplementary Fig. 2b, the distribution of O and C elements is uniform on the surfaces and interfaces of the liquid metal droplets. This indicates that the liquid metal droplets are evenly dispersed in the Carbopol gel, and the emulsion structure exhibits good stability. After drying, the Carbopol adsorbing on the surface of the liquid metal droplets separates the droplets from each other.

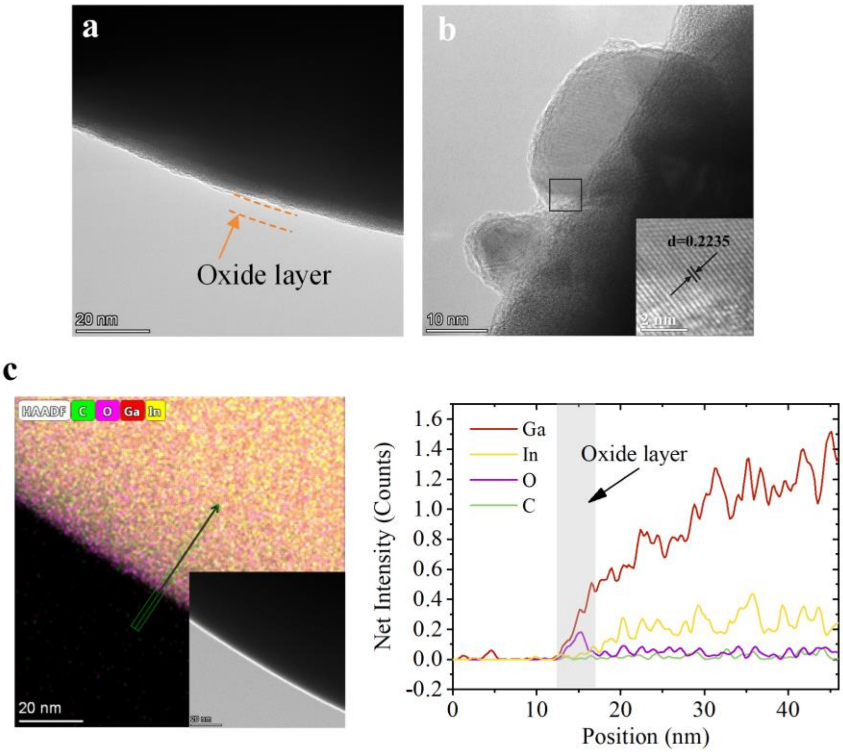

Supplementary Fig. 3 ∣ a-b TEM images of liquid metal droplets dispersed in ethanol. **c** TEM/EDS line scan moves from the surface of the liquid metal droplet towards the center.

Supplementary Fig. 3 shows TEM images of liquid metal droplets dispersed in ethanol. Supplementary Fig. 3a shows the thickness of the oxide layer is 5 nm. By utilizing Digital Micrograph software to calculate the interplanar spacing as 0.2235 nm (Supplementary Fig. 3b), which corresponds to the (200) planes of Ga_2O_3, the presence of surface gallium oxide is thereby demonstrated. Based on the density of the element distribution (Supplementary Fig. 3c), the oxide layer is estimated to be about 5 nm thick, which also confirms that the main component of the oxide layer is gallium oxide.

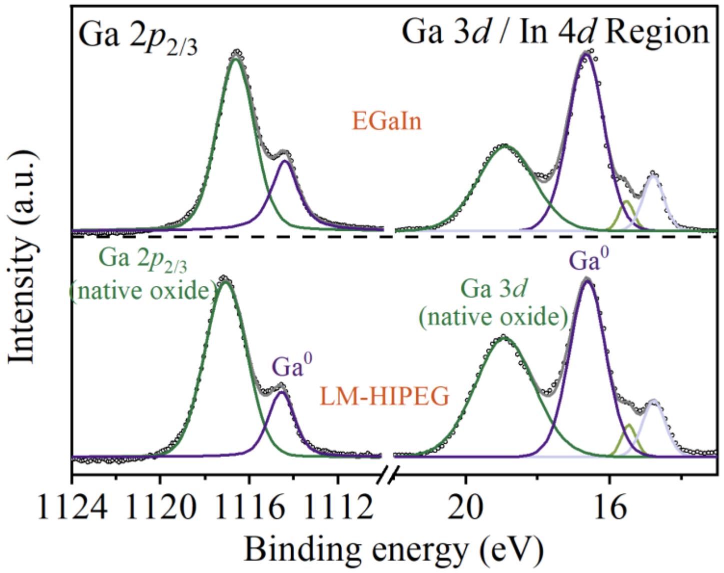

**Supplementary Fig. 4 | ** XPS spectra of LM-HIPEG.

This layer is believed to be composed of amorphous oxide. X-ray Photoelectron Spectroscopy (XPS) analysis (Supplementary Fig. 4) reveals changes in the oxidation states of Ga before and after the formation of LM-HIPEG. In the pure LM, Ga3/2*p* exhibits two distinct peaks corresponding to Ga^{3+}/Ga^{2+} states (binding energy = 1116.5 eV) and a Ga^0 state (binding energy = 1114.4 eV). The Ga3*d* line (overlapping with In4*d*) also represents Ga^{3+}/Ga^{2+} states (binding energy = 18.9 eV) and a Ga0 state (binding energy = 16.7 eV). Following the formation of LM-HIPEG, there is an increase in the area ratio of peaks associated with the Ga^{3+}/Ga^{2+} states in both Ga4d and Ga3/2*p* lines, indicating that there is a higher degree of Ga oxidation occurring. In comparison to gallium, indium is less prone to oxidation[1], and the In4*d* line in the spectrum does not show a significant change, suggesting that the surface layer on EGaIn droplets is primarily composed of gallium oxide.

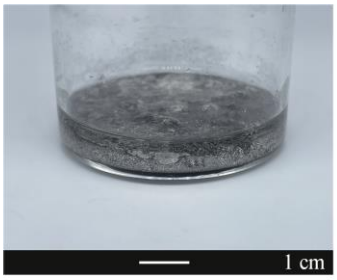

**Supplementary Fig. 5 | ** State of liquid metal after shearing in water in an air environment.

In the air environment, although continuous shearing liquid metal can generate the oxide layer and disperse it into small droplets, the droplets sink onto the bottom of the flask due to the high density of the liquid metal, causing rapid droplet coalescence.

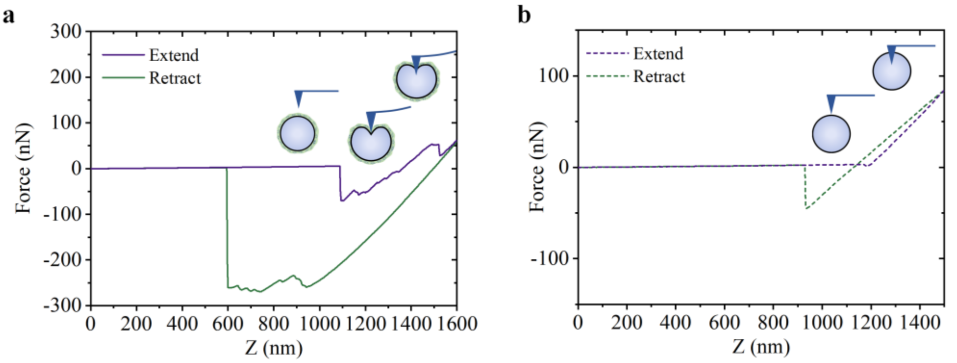

Supplementary Fig. 6 | ** The force curves of AFM tip pressing on EGaIn droplets. **a The force curves on a EGaIn droplet with an oxide layer and a hydrogel layer. **b** force curves on an EGaIn droplet with only an oxide layer.

We quantified the breakthrough of EGaIn droplets (diameter ≈ 40 μm) coated with a Carbopol hydrogel layer under external forces using force-displacement measurements with an atomic force microscope (AFM) (Supplementary Fig. 6a). Initially, the AFM tip is brought close to the EGaIn droplet until negative force feedback is observed, indicating attractive van der Waals forces between the tip and the hydrogel on the droplet surface. The tip-sample distance is further reduced to make contact with the surface, and gradually increasing force is applied by the tip, resulting in compression deformation of EGaIn. This is reflected in the linear force-displacement behavior. When the applied force exceeds ~50 nN, the EGaIn droplet ruptures, resulting in a kink in the force-displacement curve. During the retraction process, the force curve does not overlap with the approach curve, which can be attributed to significant adhesion between the tip and EGaIn/hydrogel. In contrast, for EGaIn droplets without a Carbopol hydrogel layer, the force curve shows an instant rupture of the oxide layer when the probe contacts the droplet (Supplementary Fig. 6b). This demonstrates that the Carbopol hydrogel layer provides a certain degree of protection for the droplets.

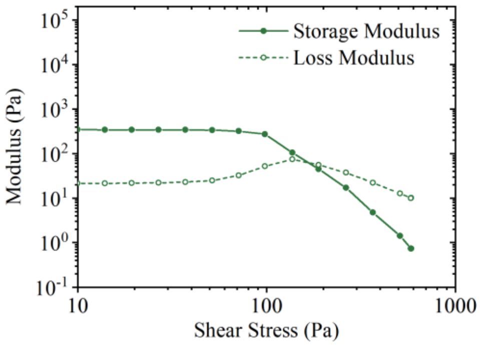

Supplementary Fig. 7 ┃ Modulus as a function of shear stress of Carbopol hydrogel with a mass fraction of 0.67%.

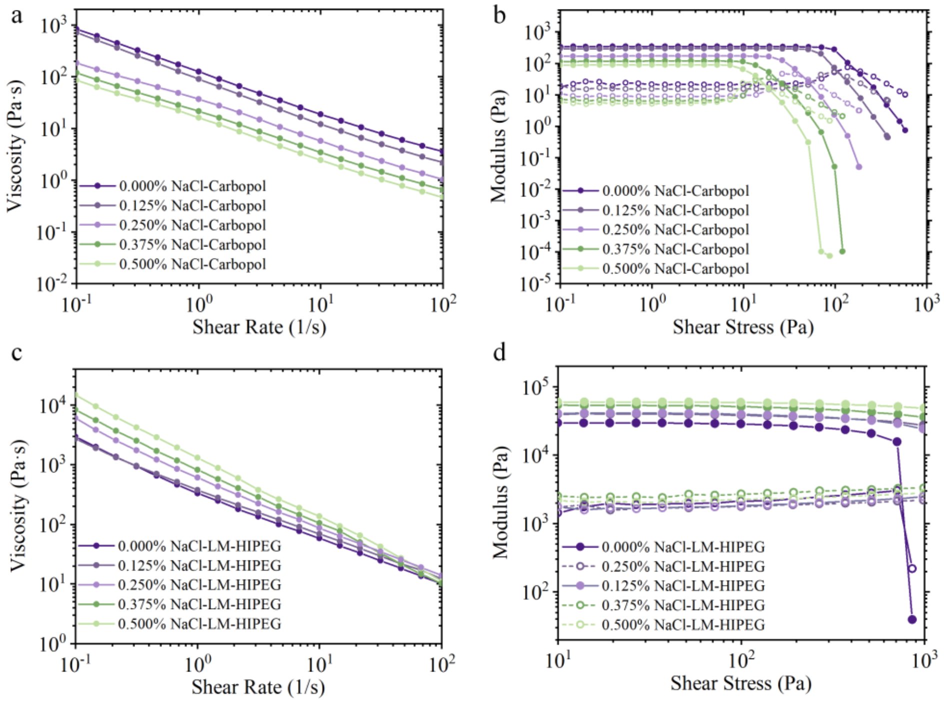

Supplementary Fig. 8 | ** The impact of ion concentration on the rheological behavior of Carbopol hydrogels and LM-HIPEG. **a Viscosity as a function of shear rate of Carbopol hydrogel with varying mass fractions of NaCl. **b** Modulus as a function of shear stress of Carbopol hydrogel with varying mass fractions of NaCl. **c** Viscosity as a function of shear rate of LM-HIPEG with varying mass fractions of NaCl. **d** Modulus as a function of shear stress of LM-HIPEG with varying mass fractions of NaCl.

Adding sodium chloride to Carbopol hydrogel will cause the viscosity and elastic modulus of Carbopol hydrogel to decrease (Supplementary Fig. 8a-b). However, The liquid metal can still be dispersed into it, and the modulus of the obtained ink increases slightly (Supplementary Fig. 8c-d), possibly because the addition of ions reduces the interfacial tension between the liquid metal and the gel, making the dispersed droplet smaller. This result clearly demonstrates that the rheological properties of the ink are not determined by the modulus of the gel matrix.

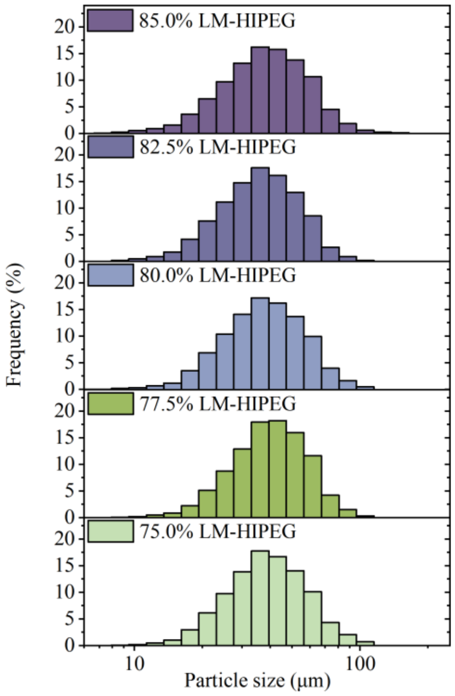

**Supplementary Fig. 9 | ** Droplet size distribution of LM-HIPEG with different volume fractions of EGaIn (75% ~ 85%).

The size distribution of EGaIn droplets tends to remain consistent across different volume fractions of LM-HIPEG after prolonged shear dispersion. The LM droplet size distribution has a maximum of 35 μm. A small number of large droplets are close to 100 μm, and also some other droplets are smaller than 10 μm.

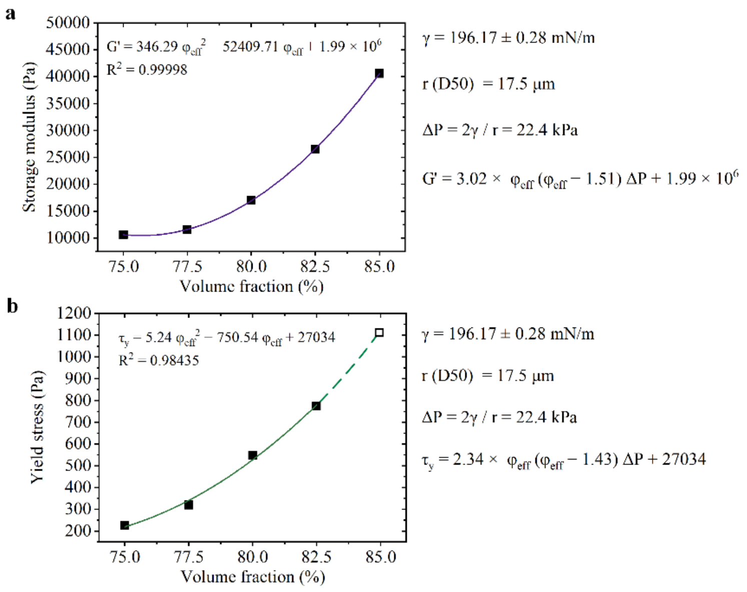

Supplementary Fig. 10 | The relationships between the EGaIn volume fraction and both the energy storage modulus and the yield stress. **a** Nonlinear fitting of the volume of EGaIn and the storage modulus(G') of the ink. **b** Nonlinear fitting graph of the volume of EGaIn and the yield stress(τ_y) of the ink.

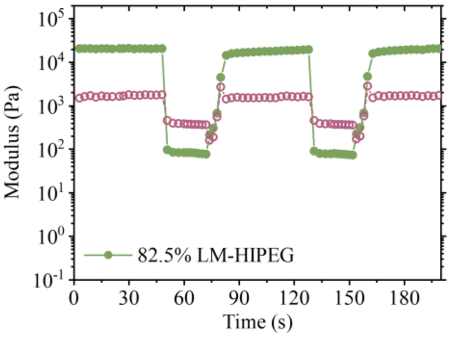

Supplementary Fig. 11 | The 3ITT curve of 82.5% LM-HIPEG. Structural restoration under low shear strain conditions (amplitude strain = 0.01%) for 20 s; structural deformation under high shear conditions (amplitude strain = 200%) for 60 s.

Supplementary Fig. 11 shows the 3ITT results for 82.5% LM-HIPEG, indicating that the ink can recover 75% of its structural strength within 15s and more than 95% within 35s.

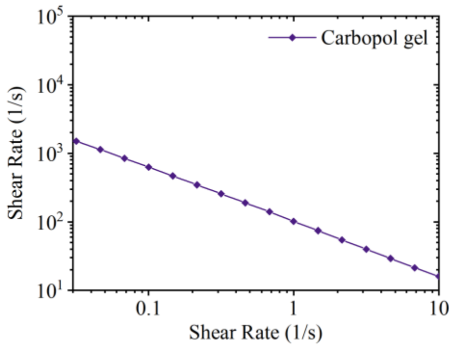

Supplementary Fig. 12 | Viscosity as a function of shear rate of Carbopol hydrogel with a mass fraction of 0.67%.

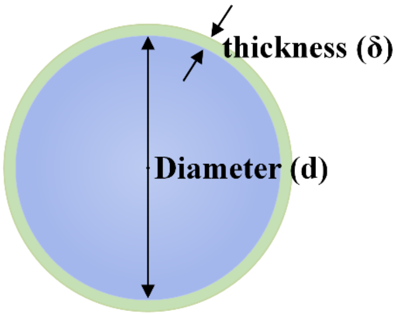

Supplementary Fig. 13 │ Quantify Carbopol Thickness.

In the LM-HIPEG, the liquid metal droplets are approximated as spheres, each coated with a uniform Carbopol hydrogel layer, as depicted in the schematic below. Based on the volume ratio of liquid metal to hydrogel and the particle size distribution graphs, the thickness of the Carbopol hydrogel layers in 75%, 77.5%, 80%, 82.5%, and 85% LM-HIPEG can be calculated as 2.4, 2.1, 1.8, 1.6, and 1.3 μm, respectively. The calculation process is as follows:

$$\sum \frac{4}{3}\pi n_i\left(\frac{d_i}{2}\right)^3 : \sum \frac{4}{3}\pi n_i\left[\left(\frac{d_i}{2}+\delta\right)^3-\left(\frac{d_i}{2}\right)^3\right]=V_{\mathrm{EGaIn}}:V_{\mathrm{Hydrogel}}$$

Here, n_i and d_i represent the frequency and particle size, respectively, in the particle size distribution graph. δ denotes the thickness of hydrogel, and V represents volume.

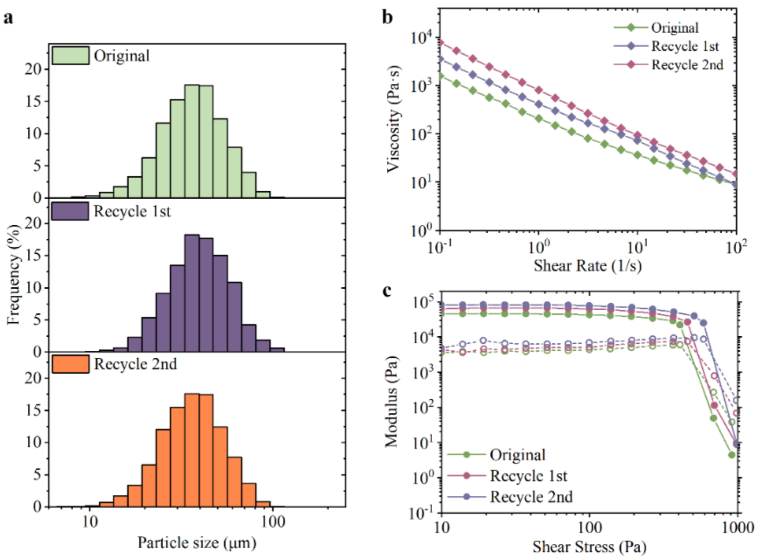

Supplementary Fig. 14 | ** Properties of the 82.5% LM-HIPEG ink after extrusion. **a Particle size distribution of 82.5% LM-HIPEG in its original state, after the first recycling, and after the second recycling. **b** Viscosity as a function of shear rate for LM-HIPEG(original recycle 1st, and recycle 2nd). **c** Modulus as a function of shear stress for LM-HIPEG(original recycle 1st, and recycle 2nd).

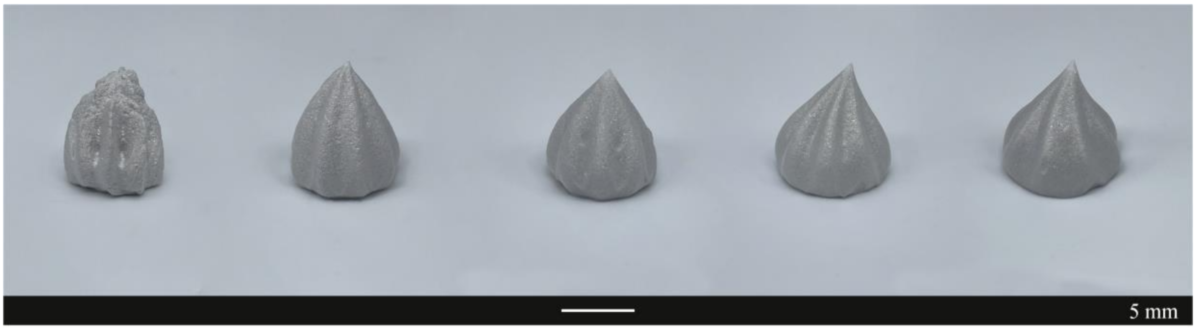

Supplementary Fig. 15 │ Images of LM-HIPEG inks with a volume fraction of liquid metal ranging from 85% to 75%.

From Supplementary Fig. 15, it can be observed that as the volume fraction of liquid metal increases, the ink exhibits better self-supporting behavior. The ink with 85% volume fraction has a high viscosity, making it difficult to extrude from a 200 μm needle. On the other hand, the ink with 75% volume fraction shows slight creep behavior. To achieve the best printing results, in this work, we used ink with a volume fraction of 82.5%.

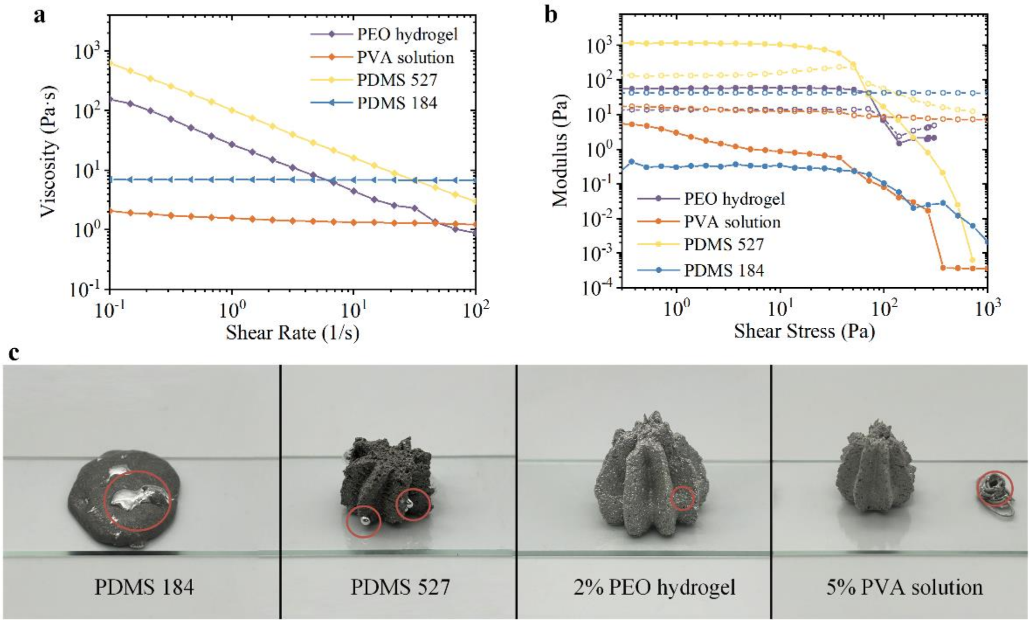

Supplementary Fig. 16 │ Compared to common polymer materials. **a** Viscosity as a function of shear rate of 2% PEO hydrogel, 5% PVA solution, PDMS 527, PDMS184. **b** Modulus as a function of shear stress of PEO hydrogel, PVA solution, PDMS 527, PDMS184. **c** Images of composite materials composed of EGaIn and common polymer materials.

EGaIn struggles to form uniform droplets within PDMS 184 at a high volume fraction of 82.5%, because of PDMS 184's high viscosity. In contrast, PDMS 527 has a much lower viscosity than PDMS 184 and can mimic the rheological behavior of Carbopol hydrogel by adding PTFE. However, we observed obvious demulsification when EGaIn is dispersed in PDMS 527 and subjected to continuous shear force. This issue arises because the droplet surfaces lack a lubricating layer, and the shear force breaks the oxide layer due to the movement of polydimethylsiloxane molecules. Additionally, the lack of carboxyl groups in PEO (polyethylene Oxide) and PVA (polyvinyl Alcohol) molecules limits their interaction with the oxide layer of the dispersed EGaIn. This interaction is much weaker than the interaction between carboxylate ions and Ga^{3+}. As a result, the dispersion of EGaIn in PEO hydrogels and PVA solutions is uneven, and some visible metallic lusters are found on the ink surface. The severe

demulsification observed when extruded through a needle further proves the instability of the droplet dispersion.

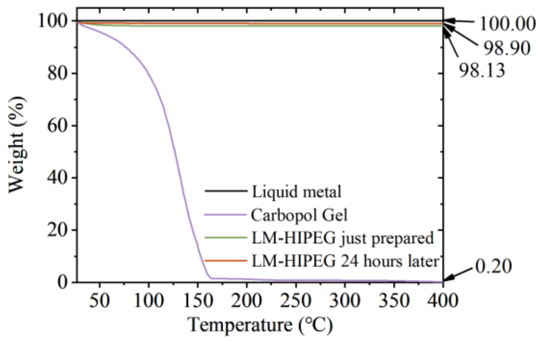

Supplementary Fig.17 | The thermogravimetric curve of LM-HIPEG.

From Supplementary Fig. 17, it can be inferred that the initial mass fraction of water in as-prepared LM-HIPEG is 1.67%. After 24 hours, the mass fraction of water decreases to 0.90%. This indicates that the surface drying of LM-HIPEG leads to close packing of EGaIn droplets, which hinders the evaporation of internal water and is beneficial for maintaining the 3D structure.

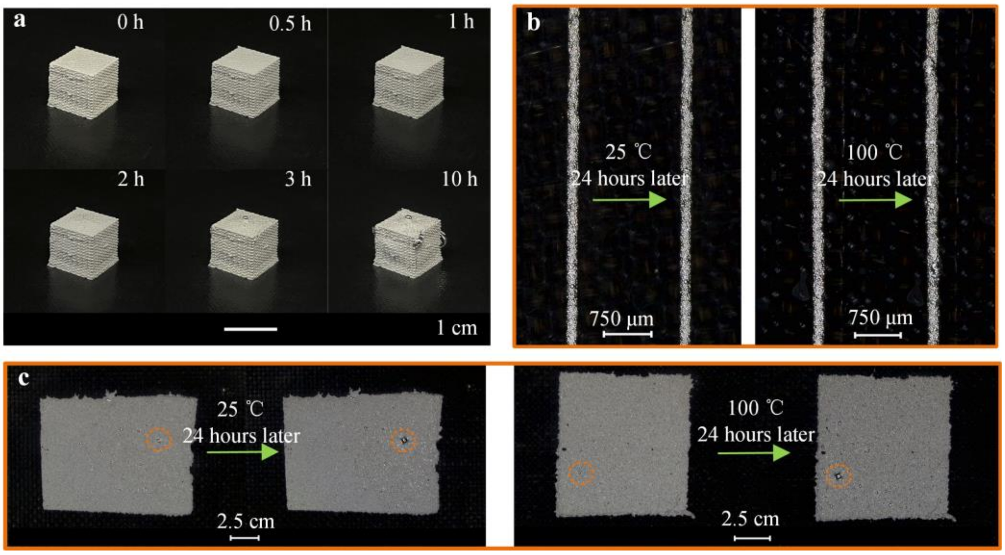

Supplementary Fig. 18 | Morphological changes of printed objects over time. **a** The printed cube 3D object under ambient conditions. **b** The printed line at 25°C and 100°C. **c** The printed 2D pattern at 25°C and 100°C.

As shown in Supplementary Fig. 18a, the printed small cube 3D object can maintain its stable shape within the first two hours. After three hours, some large EGaIn drops begin to appear from the object, indicating the rapture of the oxide layer in the ink. The printed lines and 2D patterns exhibit better shape retention. As shown in Supplementary Fig. 18b, after 24 hours, the shapes of 2D patterns and lines do not significantly change, even in ambient conditions or at 100°C. There is no extensive merging of liquid metal droplets. In fact, the easy activation of the ink is due to the easy merging of liquid metal droplets, which also results in shape change. Therefore, even in 2D structures, the ink must be encapsulated within certain materials and cannot be directly exposed to the environment.

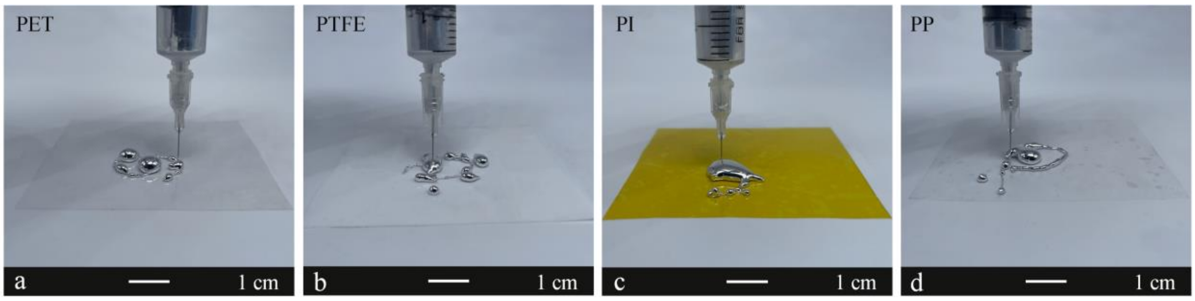

Supplementary Fig. 19 │ Images of EGaIn droplets on different polymeric substrates. **a** PET. **b** PTFE. **c** PI. **d** PP.

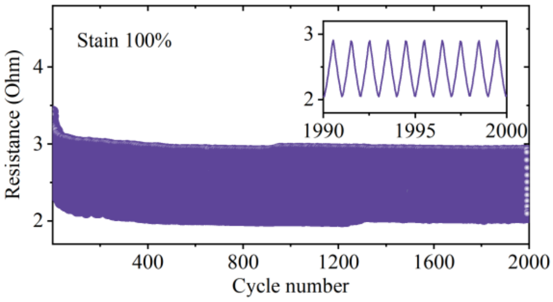

Supplementary Fig. 20 │ The 2000 cycles of resistance variation in a printed line under 100% deformation.

The resistance of LM-HIPEG ink shows a linear variation with strain. The printed line also exhibits outstanding cyclic stability, showcasing its potential to be used as a flexible strain sensor.

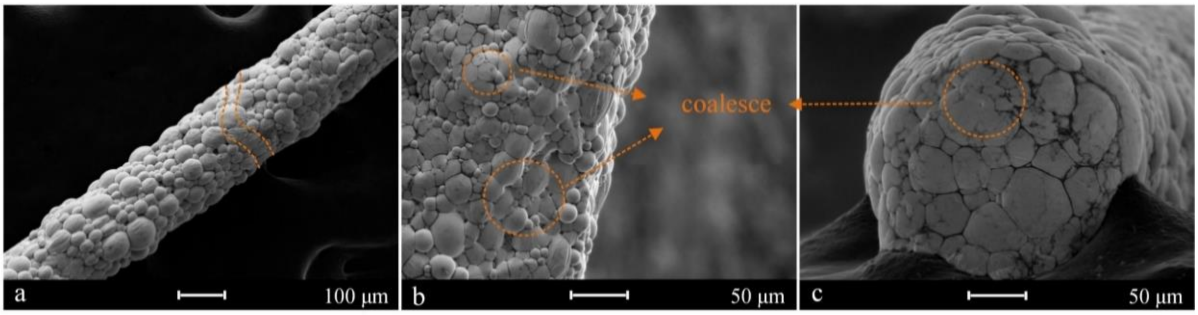

Supplementary Fig. 21 | SEM images of LM-HIPEG after solidification. **a** The surface of a printed line. **b** The surface of the bulk sample. **c** The cross-section of the printed line.

As shown in Supplementary Fig. 21a, compared to Fig. 3a, the droplets on the surface of the line-shaped sample transitioned from smooth to wrinkled. This is because the overall crystalline volume of the line-shaped sample expands. Once the ambient temperature returns to room temperature, the droplets revert to liquid droplets, and a new oxide film rapidly forms on the surface, resulting in the restoration of the sample volume and the formation of wrinkled surface structures. From the surface view (Supplementary Fig. 21b), it can be observed that some droplets, due to volume expansion, coalesce and develop into larger polygonal droplets, creating a conductive pathway.

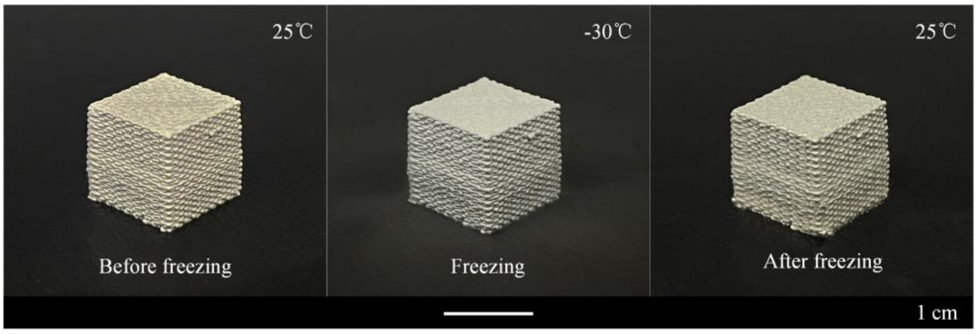

Supplementary Fig. 22 │ Shapes of the printed cube before, during, and after solidification.

From Supplementary Fig. 22, it can be observed that there are no significant changes in the macroscopic shape of the printed cube before, during, and after freezing.

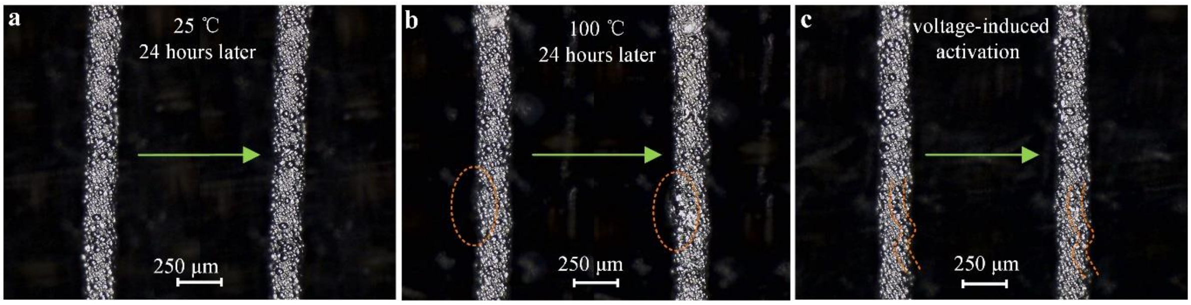

Supplementary Fig. 23 | ** The merging of droplets on printed lines. **a At 25°C after 24 hours. **b** At 100°C after 24 hours. **c** After voltage-induced activation.

We placed the printed lines at 100°C for 24 hours, as shown in Supplementary Fig. 23. Only a small portion of the droplets on the line merged due to thermal expansion. Thus, after 24 hours at 100°C, some lines became conductive while others did not. The droplet merging on the voltage-induced activated line is rapid and complete, all lines activated under voltage induction.

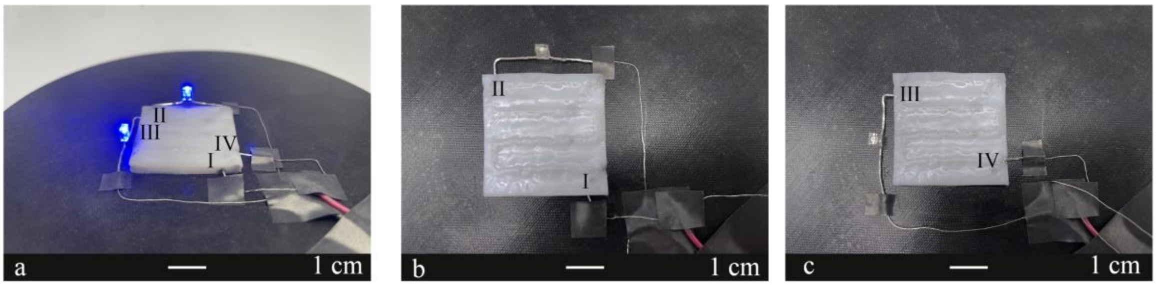

Supplementary Fig.24 | 3D network formed by alternating printing of LM-HIPEG with PDMS/PTFE. **a** Connecting I with III and II with IV of the circuit. **b** connecting I with II of the circuit. **c** Connecting III with IV of the circuit.

As shown in Supplementary Fig. 24a, connecting I with III and II with IV can both achieve electrical conduction. However, as shown in Supplementary Fig. 24b and c, terminals I and II are disconnected, and so are terminals III and IV. These results are consistent with the design and demonstrate that LM-HIPEG can be utilized in printing 3D circuits.

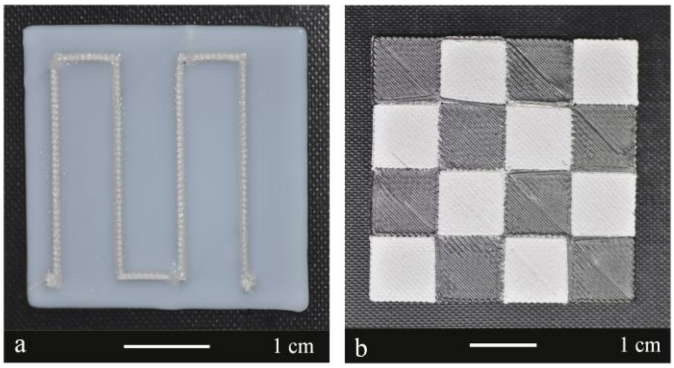

Supplementary Fig. 25 | Top-view of the objects printed with alternating materials. **a** Object printed with LM-HIPEG and PDMS/PTFE **b** Object printed with LM-HIPEG and EP/GN.

Supplementary References

1. Tostmann, H. et al. Surface structure of liquid metals and the effect of capillary waves: X-ray studies on liquid indium. *Phys. Rev. B* **59**, 783-791 (1999).
