# Supplementary material for: High internal phase emulsions gel ink for direct-ink-writing 3D printing of liquid metal
Source: Nat Commun. 2024 Jun 5;15:4806. doi: 10.1038/s41467-024-48906-w (PMC11153652; doi:10.1038/s41467-024-48906-w)
Supplement: Supplementary file 2 — Description of Additional Supplementary Files [file 41467_2024_48906_MOESM2_ESM.pdf]

Description of Additional Supplementary Files

Supplementary Movie 1: 3D printing of LM-HIPEG at a speed of 30 mm s^{-1}.

Supplementary Movie 2: Activating circuits printed on PDMS by stretching.

Supplementary Movie 3: Activating circuits by cryogenic freeze crystallization.

Supplementary Movie 4: The electrocapillary effect of EGaIn in Carbopol gel. Activating a printed line with a 21 V voltage. Morphological changes of the line when activated under the microscope.

Supplementary Movie 5: Alternating printing of LM-HIPEG with PDMS/PTFE.

Supplementary Movie 6: Extrusion speed of LM-HIPEG under different air pressures. Printing conditions of LM-HIPEG at different printing speeds.
